# Advanced therapy medicinal products: value judgement and ethical evaluation in health technology assessment

**DOI:** 10.1007/s10198-019-01147-x

**Published:** 2020-01-09

**Authors:** Elisabete Gonçalves

**Affiliations:** Department of HTA and Market Access, Real World and Late Phase, CTI Clinical Trial & Consulting, Lisboa, Portugal

**Keywords:** Advanced therapy, Health technology assessment, Value, Ethics, Methods

## Abstract

Advanced therapy medicinal products (ATMPs) are a heterogeneous class of medicinal products that by offering the potential of cure represent a paradigm shift in the approach of many life-threatening diseases. Although a common regulatory framework for ATMPs has been established in the EU, the health technology assessment (HTA) and financing decisions remain local. The aim of this article is to present an integrated analysis of the current status of the value judgment of ATMPs and the integration of ethical evaluation in the HTA process. It has been identified that approaching the specificities of ATMPs in terms of market access will require a broadening of the definition of value to be able to systematically capture elements of value not traditionally considered. Outcomes modelling will play an important role in the pricing and reimbursement of ATMPs, providing a way to bridge the gap caused by the absence of data from clinical studies or real-world data. Given the nature and disruptive consequences of ATMPs the assessment and adoption of these medicinal products raises important ethical questions, both at a policy and at society level that should be properly addressed. HTA can be made more transparent and reliable, and simultaneously promote robust and accountable decision making, by turning explicit the value judgments implicit in HTA. Ultimately, there should be no core conflict between ethical requirements and HTA in a scenario where the goal is to promote equity and access of patients to truly innovative therapies such as ATMPs, while assuring the sustainability of healthcare systems.

## ATMPs: definition and regulation

Advanced therapy medicinal products (ATMPs), are a heterogeneous class of medicinal products that includes products based on genes (gene therapy medical products), cells (somatic cell therapy medical products) and tissues (tissue engineering medical products) [1]. ATMPs may offer the potential of a one-time cure and are considered disruptive technologies [2].

These novel highly complex therapies need to be addressed differently when compared to traditional medicinal or biologic products in their development, manufacturing, or administration process, with emphasis in the establishment of batch consistency, product stability and product safety and efficacy through pre-clinical and clinical studies [3].

Particularly, they are also expected to bear a higher risk potential compared to other biological medicinal products such as tumorigenicity, cell differentiation and patient integration [3].

A legal framework has been established in the European Union (EU) governing the regulation of all medicinal products for human use, including ATMPs. A consolidated regulatory framework for ATMPs was introduced by Regulation 1394/2007/EC of the European Parliament and of the Council of 13 November 2007 on ATMPs and amending Directive 2001/83/EC and Regulation 726/2004/EC. This regulation provides specific regulatory principles for the evaluation and authorization of ATMPs and filled a pre-existing regulatory gap by subjecting these innovative products to the general EU pharmaceutical legislation [3, 4].

The ATMPs regulation defines three specific types of medicinal products, including gene therapy medicinal products, somatic cell therapy medicinal products, and tissue-engineered products, all of which meet one of the definitions of medicinal products established [4]. Additionally, combined ATMPs contain a medical device, as an integral part of a viable cell- or tissue-containing product, or non-viable cells or tissues, which are liable to act upon the body with an action that can be considered primary to the device [4]. A Committee for the Advanced Therapies (CAT) was established and is responsible for all regulatory procedures concerning ATMPs in the EU [5]. In the EU regulators have also developed specific pathways, including EMA (European Medicines Agency) Priority Medicines scheme (PRIME), conditional approval, exceptional circumstances, accelerated assessment and compassionate use to promote faster approval of promising new therapies, such as ATMPs [6].

ATMPs often target rare diseases and can be designated as orphan medicinal products in the EU. In this region, in order for a product to qualify for orphan designation, the prevalence of the disease addressed must not be superior to 5 in 10,000 people [7].

## HTA of ATMPs in the Big 5EU

In the ATMPs product development, to achieve the market access, developers need to first have a clear understanding of the markets and their reimbursement requirements before initiating clinical studies, namely to have a clear understanding of the market environment for the target indication, the disease burden, the target patient type, the alternative treatments already available, the reimbursement profiles of alternative products and the reimbursement policy of the relevant payers [8]. Additionally, the minimum efficacy thresholds necessary to support a commercially viable reimbursed price, will need to be the identified [9]. In summary, to achieve success in the HTA and market access strategy, it will be essential to understand whether there is room for accommodating the innovation and to establish a solid positioning strategy [8].

In the EU the marketing authorization for a new medicine via the centralized procedure involves a series of quality and clinical reviews by the EMA. Following marketing authorization, HTA bodies or payers on a country-by-country basis will undertake a review of the clinical evidence prior to conducting the economic review. While for the EMA the focus of the clinical review is assessing the quality, efficacy and safety of the product, the clinical review undertaken by HTA bodies/payers will focus on assessing comparative effectiveness against an existing therapeutic alternative or the best supportive care. The size of the incremental clinical benefit acknowledged is then used as a basis for the economic analysis, together with cost considerations. Finally, before patients can have access to the new therapy, a decision to include the therapy in any formulary is usually made at the local level, is subject to budget impact considerations, and may change periodically according to budget cycles [8].

Despite the centralization of the marketing authorization process, the reimbursement process is greatly fragmented across countries and even across regions of a given country. Therefore, there are significant variations in pricing and reimbursement frameworks among the five largest EU countries (Germany, United Kingdom, France, Spain and Italy), that is, the Big 5EU [8].

The EUnetHTA (European Network for Health Technology Assessment) HTA definition states that: “Health technology assessment is a multidisciplinary process that summarizes information about the medical, social, economic and ethical issues related to the use of a health technology in a systematic, transparent, unbiased, robust manner. Its aim is to inform the formulation of safe, effective, health policies that are patient focused and seek to achieve best value” [10].

Currently, the HTA procedure is considered a standard policy tool for informing decision makers who manage the entry of new medicinal products, medical devices, and other technologies in health systems, namely, through pricing and reimbursement [11].

However, individual EU HTA agencies have adopted different priorities and methods, linked to their health system funding model and the weighting of economic/budget impact versus broader clinical or societal impact. This variation across HTA decision making is perhaps more visible when considering access to ATMPs than to conventional therapies. Some HTA bodies show more willingness than others to admit new types of evidence beyond randomized controlled trials, or to consider economic models that include extrapolating longer-term benefit from limited existing data.

Affordability is vital to the sustainability of healthcare systems, however, budget impact analysis can pose a challenge to rewarding and promoting innovation such as ATMPs. On one hand, the focus of the analysis is typically the healthcare budget isolated, and savings in the social care budget (e.g., rehabilitation or long-term social care) are not adequately captured in the benefits assessment. On the other hand, the time horizon of the analysis is often 1–2 years, which implies that long-term benefits, such as in the case of ATMPs, are also inadequately captured [9].

Over the years, the assessment of price and reimbursement for innovative therapies has shifted towards value-based models [8, 9]. However, the methods by which added value is captured and translated into a reimbursed price vary in different geographies. The frameworks used by EU HTA bodies are typically based on comparative clinical benefit assessment and economic evaluation (cost utility analysis or cost-effectiveness analysis) as the key methods for defining the value of new technologies. This may involve reflecting health gain as quality-adjusted life years (QALY), or an alternative patient-relevant outcome, such as life years gained (LYG) [2].

Jørgensen and colleagues identified the pricing, reimbursement and market access considerations most relevant for ATMPs in the Big 5EU. The clinical benefit is measured in comparison to a suitable comparator. This comparator can differ between countries, since clinical practice may be different in different settings, which raises the need to perform indirect comparisons [9].

In the Big 5EU, the only country that links the cost per QALY specifically and methodologically to pricing and reimbursement is the United Kingdom. In Germany, cost-effectiveness analysis is not commonly used, which means comparative efficacy and budget impact remain the main decision-making criteria for pricing and reimbursement [9].

Other factors, such as the contribution to gross domestic product (GDP), involvement of patient advocacy groups as well as ethical, equality, and equity considerations can also impact on the final decision on a country level [9].

Despite their significant therapeutic potential, ATMPs face specific pricing and reimbursement challenges. ATMPs have high manufacturing costs, which will demand a high target price to achieve commercial viability. Their incremental benefit claims can cover a longer horizon than is supported by clinical trial data at launch since ATMPs may potentially provide life-long benefits.

Also, ATMPs require complex interventional procedures for their administration and are therefore likely to be restricted to centers of excellence [9]. Another potential barrier identified for ATMPs implementation is that some of these treatments change the patient pathway, and the means treatments are delivered. Consequently, the ability of national health service organizations to adapt existing infrastructure and care delivery can present additional hurdles to the adoption of ATMPs [9].

Often clinical studies evaluating ATMPs may also be more dependent on surrogate outcomes instead of clinical outcomes, which poses additional challenges to the assessment [9].

Outcomes modelling will play an important role in the pricing and reimbursement of ATMPs. In therapeutic areas with a high unmet need it may be considered not ethically justifiable to randomize patients to receive placebo as a control, when an efficacious alternative is already available. In these cases, single-arm trials may be performed, which provide a lower grade evidence from the payer perspective, as they only allow an indirect comparison with the standard of care, which adds additional challenges to the pricing and reimbursement negotiations. Additionally, the timeframe of a clinical trial does not allow to capture long-term benefits, which means that an important part of the value proposition is not documented, such as in the case of the curative treatments of some ATMPs.

Outcomes modelling is a way to bridge the gap caused by the absence of satisfactory data from clinical studies or real-world data. Therefore, for many ATMPs, indirect comparisons and extrapolations become particularly relevant. They allow data comparison from different sources where direct comparisons are unavailable. Indirect comparison is particularly relevant for ATMPs in two situations: in cases where the comparator in the pivotal trial does not reflect the standard of care in the country in question, or in cases where ethical considerations demand the performance of a single-arm study. This methodology offers a way to use data from other studies or observational sources such as meta analyses and registries to estimate the comparative effectiveness of the new treatment [9].

In terms of market access solutions, risk sharing agreements between the manufacturers and payers are a way to reduce the uncertainty related with the lack of long-term data at launch. It is important to note that these schemes require regular patient follow-up and are often associated with significant clinical and administrative burden, which has limited their implementation [9].

Ultimately, the demonstration of effectiveness and comparative effectiveness of ATMPs in the long term will need to be complemented by the collection and subsequent analysis of real-world evidence [2].

## Redefining value for ATMPs

In a market economy, the value of an economic good can be regarded in two ways: what individuals are willing to pay for it (a demand-side view) or what individuals have to trade off to obtain it (an opportunity cost or supply-side view) [12]. Currently, there is no consensus on how to appropriately reward the value of the innovation brought by orphan drugs and ATMPs [12].

Garrison and colleagues characterized the challenges for traditional approaches in assessing the value of one-time gene replacement therapies and proposed a health economic rationale for a higher value-based cost-effectiveness threshold (CET). It is generally recognized that ultrarare, health-catastrophic conditions should be assessed against a higher CET [12].

The administration of a one-time dosing with potential lifelong benefit creates new challenges for payers to adequately reward the manufacturers of such innovations with an adequate return on their investment. The measured value and financing of pharmaceutical products have been traditionally strongly influenced by the duration of treatment. Economic value is usually defined in terms of health gained, that is QALY plus cost-offsets evaluated over the defined time horizon. Yet, conveying appropriate value in the long term for one-time therapies is problematic since this usually requires extrapolation from small clinical trials of short duration [12].

The ICER (Institute for Clinical and Economic Review) has debated a range of up to $500K per QALY for ultra rare diseases but has informed it will still publicize the base-case value-based price at a CET of $150K per QALY gained. In the United Kingdom, the National Institute for Health and Care Excellence (NICE) has defined gene therapies as “highly specialized technologies” that should be subject to a much higher, yet variable, threshold up to £300K ($390K) per QALY depending on the magnitude of the QALY gains [12].

A recent special task force (STF) of the International Society for Pharmacoeconomics and Outcomes Research (ISPOR) specified, “Health plan coverage and reimbursement decisions should consider cost-effectiveness analyses, as measured by cost per QALY, as a starting point”. It further recommended that elements of costs and benefits not usually included in cost-effectiveness analysis that affect individual well-being (such as severity of illness, equity, and risk protection) may be relevant for some health plan decisions. Still, more research is needed on how best to measure and include them in decision making [12].

The potential elements of value identified in the ISPOR STF, in this broader perspective were divided into three categories: (1) core elements of value (QALY, net cost); (2) common but consistently used elements of value (productivity, adherence-improving factors); (3) potential novel elements of value (value of knowing, fear of contagion, insurance value, severity of disease, value of hope, real option value, equity and scientific spillovers) [12].

Jena and Lakdawalla reinforced the importance of several other elements in this assessment: health equity (related to severity of disease), caregiver burden, and family spillovers (in terms of the negative effect on the well-being of family members) [12].

Jönsson and colleagues also proposed that these wider benefits should include disease severity, age of onset, lifetime burden of illness, socioeconomic impact, and possible spillovers from the initial innovation [2].

It seems clear that value will need to be considered differently for ATMPs. Additional elements of value (beyond health gain) should then be considered in the evaluation of these products [2].

## ATMPs: general ethical concerns

“Ethics is nothing but a technology to make a particular set of (potential) problems manageable and controllable” [13]. Bioethics specifically is a primarily interdisciplinary field of inquiry that engages scholars from disciplines such as health sciences, philosophy, law, economics and sociology. These different scholars draw on different theoretical approaches and often will use both qualitative and quantitative methods to conduct evidence-based analyses in the fields of biology, medicine and others [14]. The relationship between the natural sciences and ethics has been an often debated topic in modern philosophy and has been addressed in several ways [15]. It is commonly accepted that decision making in medicine, research, and health policy often explicitly or implicitly considers normative ethical aspects [16]. Considering a sociological perspective, ethics can be described as the product of society and social norms and values that will act as the basis of ethical evaluation and ethical reasoning [10].

The fundamental principles of bioethics and biolaw include the respect for life in all its forms, and the quality of the environment, to ensure the maintenance of life and vital processes, with a constant commitment to transparency and the spreading of knowledge involving biological and medical sciences [17].

Thomas Beauchamp and James Childress proposed in the book Principles of Biomedical Ethics, Principlism and its four principles. These principles are: autonomy, beneficence, non-maleficence and justice. The key characteristic of this model is the equal value of each principle: they are always valid and binding unless they are in conflict and it is not established if one takes priority over the others [18].

Regarding ATMPs, though genome editing consists of a general process, it is important to highlight that the ethical implications of gene editing are not just about the process, but instead are directly related to the purpose for which it is used [19].

From the perspective of bioethics, the use of gene therapy appears to be closely related to several challenging factors, such as economic difficulties, particularly with regards to wealth distribution, political and cultural conflicts, as well as the scarcity of studies evaluating the impacts of the use of gene therapy on human health. These aspects raise both clinical dilemmas and legal issues [17].

In general, clinical research on genetic diseases raises scientific and ethical concerns, that include specific ethical procedures for genetic research, collection, storage and access to genetic materials, aims of the use of genetic information, the definition of the time of archiving genetic material in biobanks, informed consent and confidentiality issues and concerns specific of pediatrics [20].

In terms of marketing authorization of ATPMs, the fact that conditional approval takes place at a stage that is still part of the research phase adds complexity to the ethical questions raised [21].

Also, in the case of ATMPs, when a product is autologous (originated from the same patient), the question of the ownership of the product can be posed, namely whether the developer or the patient are the righteous owners of the product [20].

When pediatric patients are affected by rare diseases, difficulties double as children should be considered ‘twice orphan’, and randomized controlled trials, considered as the standard in research design, are even more unfeasible due to the smaller number of patients. It becomes ethically problematic to propose a control arm (in which the investigational product/approach is not provided to a segment of the population) for a study aimed to establish the efficacy of a new product [20].

When considering ethics in the scope of gene therapy, the concept of changing the DNA of an individual, even to cure a fatal genetic disease, differs from more traditional therapeutic solutions such as surgery, medicinal products, among others, and can raise several concerns [22].

Hunt proposed in 2008 a list of topics for discussion (Table 1).

**Table 1:** Topics of discussion in gene therapy ethics, adapted from Hunt, 2008 [22]

What criteria should be met before proving that an experimental therapy is safe enough and ethical enough to begin investigating in humans?
Is the scientific review process sufficient? What constitutes adequate oversight of gene therapy trials?
Are protocol guidelines in the best interest of research study participants?
Who decides which traits are normal and which constitute a disability or disorder?
Will the high costs of gene therapy make it available only to the wealthy?
Could the widespread use of gene therapy make society less accepting of people who are different?

Currently, there are still many uncertainties about the side effects of gene therapy, despite the scientific and technological advances. Moreover, the lesser-known effects, such as long-term expression of the introduced genes, the lack of control of the expression of these genes and genetic modification of germ cells, are not known [17].

The resource to real world evidence can be considered paramount in gathering the information needed for the approval of ATMPs. In this scope, patient registries provide highly valuable but also sensitive data, and thus, demand patient privacy protections and a detailed understanding of the ethical and legal implications of proprietary use of medical data [23].

Crucial ethical issues receiving great interest by the scientific community, as demonstrated in the regulatory framework, the literature and EU projects, are genetic aspects, data protection issues, confidentiality and the handling of biological samples [20].

An important issue raised is the legitimacy of sharing patient data from different countries from the ethical point of view. This practice is increasingly widespread in the case of rare diseases and particularly challenging when these data are from pediatric patients and include genetic data. Notably, no specific ethical reference on the use of ATMPs for pediatric rare diseases is available in the current EU regulatory framework [20].

An important note is that curative therapies, such as ATMPs, have the potential to eliminate the need for long-term management and provide longer term increases in quality of life. Still, it is not known whether curative therapies are valued more highly by society than treatments that offer the same “total” health gains through marginal gains over many years and/or patients [2].

As highlighted by Rodriguez-Monguio and colleagues, the societal value of orphan drugs can be outlined by several ethical constructs. Considering that society wishes to maximize total net benefits, aligning with a classic utilitarian doctrine, aiming to maximize utility for the greatest number of individuals in society, the cost–benefit ratio of rare diseases may be positioned less favorably to receive public funding as it would in an egalitarian approach, that aims to maximize equality of all individuals. The aim of achieving an egalitarian outcome may be grounded on attaining a determined threshold of health, an overall prioritization of the worst off, or amount of resources for each individual. Therefore, an egalitarian doctrine could offer a better foundation for public funding for orphan drugs development and treatment coverage for rare diseases. Otherwise, the rule of rescue, or the capacity to intervene if a therapy becomes available, may also offer a defense for funding rare disease treatments with public funds [23].

Globally, the ethics of the resource distribution to subsidy orphan drugs has been discussed but not agreed upon in the literature. Currently there is no societal consensus on whether the size of the patient population, in itself, can act as an admissible factor in employing separate measures of effectiveness and economic evaluation (e.g., cost-effectiveness) for the approval and reimbursement of orphan drugs. Some authors argue that the special status argument for public subsidy and reimbursement of orphan drugs does not stand up to critical assessment, while others have highlighted the importance of using economic evaluation, political debate, and social dialogue to ensure distributive justice to orphan drug development and access [23].

Orphan drugs are often for treatment of life-threatening diseases, which emphasizes ethical imperatives for timely access to orphan drugs. Therefore, the right to life may be conceived as the right to health. On the other hand, the Universal Declaration of Human Rights, proclaimed by the General Assembly of the United Nations in 1948 established the right to life that was codified in the European Convention on Human Rights (1950). Additionally, the right to the “highest attainable standard of health” was first predicted in 1946 in the Constitution of the World Health Organization [23].

Considering the important specific ethical questions that are raised for orphan medicines and ATMPs, further discussion is required at a societal and policy level to assure that these questions are analyzed and taken into consideration in the policies and decisions made in terms of the HTA of these innovative medicinal products.

## Ethics applied to the HTA of ATMPs

First, it should be noted that for any new technology developed, ethical issues are raised [10].

Ethics is recognized as a crucial element in HTA since its conception, however, ethical issues are still not frequently addressed explicitly in this area [15]. Perhaps because historically, evidence-based medicine and HTA have been considered to be scientific and value-free areas [24].

According to EUnetHTA, the ethical domain involves “an understanding of the consequences of implementing or not implementing a health care technology in two respects: with regard to the prevailing societal values and with regard to the norms and values that the technology itself constructs when it is put into use” [15].

Hofmann and colleagues proposed a shift from an externalist conception of value judgments that considers value judgments as added to the results of HTA to an internalist one that recognizes and openly addresses value judgments as they arise within the HTA process [24].

In fact, the HTA process itself can raise relevant ethical issues. These include questions about the ethical consequences of the choice of endpoints or comparators, and whether there are any ethical concerns in the economic evaluation [13].

Even though the clear distinction between facts and values remains philosophically difficult to defend, it is present in the current concept and use of HTA, namely, in the distinction between assessment and appraisal, between systematic review and personal preferences, between scientific judgments and social judgments [24].

By definition HTA will include the collection and analysis of evidence from research in a systematic and reproducible way to be used for decision-making purposes by elaborating “assessment” reports [15]. “Assessment” is normally defined as the action of evaluating the relevant aspects of the health technology to reach a basis for decision making. It normally has a comparative approach: the health technology under review is evaluated compared to a standard of performance or other treatments. “Assessment” is not synonyms with “appraisal”, which usually indicates some form of recommendation regarding the implementation of the technology. The recommendation issued may lead to several actions: encouraging, discouraging or even prohibiting implementation, reimbursing, funding or disinvesting. In the EU, some HTA agencies are limited to perform assessments of the technology only and do not make recommendations about their implementation in the healthcare system. Others perform both assessment and appraisal. In line with this, the ethical analyses in HTA will vary according to the distinction between assessment and appraisal. Ethical analyses may consist in a list of ethical issues, which must be identified, described, and addressed, the most widely used modality, or, in a more complex way, moral judgements, that will classify the use of the technology in study as good/bad or licit/illicit [15].

In the end, there is no incompatibility and the appraisal process may remain deliberative, while the assessment can remain descriptive [24].

Several articles published by Hofmann and colleagues since 2005 have defended a better integration of ethical analysis in HTA [10]. HTA can be considered a process of value judgments. Nevertheless, the great number of value judgments does not make HTA biased or flawed. Values are precisely basic elements of the HTA process. Therefore, acknowledging and explicitly addressing value judgments has the potential to improve the accountability of HTA [24].

Turning explicit the value judgments implicit in HTA, for example in the framing of the research question, as well as in the appraisal stage, by weighing the information provided, can make HTA more open, transparent, and reliable, and also promote robust and accountable decision making [24].

Several value judgments are made in the economic analysis of HTA. The quantification of societal preferences for the distribution of health gains has always played a crucial role in health economics. Nevertheless, the implicitly assumed desire (preferences) to maximize health in economic evaluations may not correspond to how the majority of the people in a society think health *ought* to be distributed (values). This aspect has direct implications on resource allocation decisions.

On the other hand, cost-effectiveness analyses assume that “a QALY is a QALY,” equally weighted across individuals which consists in a quasi-egalitarian value judgment, that may be questionable in some circumstances and correcting for this aspect is not straightforward [24].

Also, the technical development of cost-effectiveness analyses also comprises important value judgments, namely through the methodological options that are made at different steps during the analysis. These relevant decision points include the choice of perspective, the target population, and the outcome measure [24].

Another important value-based choice in the economic evaluation process is related to the outcome measure used in the cost-effectiveness ratio. The LYG and QALY gained are the two most often used outcome measures. Since the valuation of health states varies between those who experience a health state directly and those who do not, consequently the outcome of the cost-effectiveness analysis will differ depending on whose values are used [24].

As Hofmann and colleagues pointed out, value judgments are greatly present in the appraisal process that will consider the outputs of the assessment, and in the decision making procedures. Today, some EU countries have created explicit processes making use of multi-stakeholder consultations. Therefore, the definition of the appraisal and the assessment-appraisal border, and likewise the organization of the appraisal process, requires a series of value judgments, such as who should perform the appraisal, who should be involved and how should stakeholders participate in the process. Also, in the appraisal process a judgment is performed on the relative importance of a series of factors that differ from appraisal to appraisal. These factors could include namely, burden of disease, effectiveness, cost-effectiveness, budget impact and extent of individual responsibility. Yet, it is important to highlight that these aspects are valued differently by the different stakeholders involved in appraisal committees, namely patient representatives, health professionals, policy makers, payers, academic researchers, industry members, care givers, citizens, etc. Different stakeholders will make different value judgments with regards to the above-mentioned aspects, which will have an impact on resource allocation [24].

A value judgment should not be understood as a “bias”, and in fact value judgments are found in all levels of what is regarded as the scientific endeavor of HTA (assessment, appraisal, and HTA-based decision making). In sum, value judgments are not at all alien to the HTA process [24].

In spite of the fact it remains uncertain how far HTA bodies should go in stating what is right or wrong, they clearly possess a central responsibility in bringing to light the diversity of socio-ethical issues that may affect individuals and society, particularly issues related to equity, transparency, social justice, and impact of technologies on marginalized groups.

The ethical principles implied suggest that decisions regarding the allocation of scarce resources must be based on fair, transparent, and nondiscriminatory criteria that enable individuals in a society with a particular health need to receive appropriate healthcare services [14].

Since there seems to be no agreement on how to better approach the crucial elements of equity, the value assumptions and judgments made during a HTA should be explicit, making transparent the ethical framework underlying the economic analysis [24].

## Ethics methodology applied to HTA of ATMPs

Contrasting with clinical and economic assessments, that aim to correctly elucidate and predict the outcomes of a technology using empirical data, ethical analysis will look for ethical values and use philosophical theories to justify reasons for implementing a technology. Hence, other approaches must be used to face ethical issues in HTA, in which HTA experts may not necessarily have specialized knowledge and skills [25].

Hofmann and colleagues developed in 2005 a checklist of thirty-three ethics questions that provided a starting point for the systematic integration of ethics into the HTA process and created the basis for developing similar sets of enquiries [26].

Notwithstanding the advances made so far in the development of ethical frameworks for HTA, there is still no clear agreement on the scope and particulars of a practical method to address ethical aspects in HTA [25].

There is a variety of theoretical approaches available for HTA bodies to analyze values, ethics, and social dimensions of new technologies. Three broad methodological approaches to the introduction of ethical and social issues into HTA reports are available: (1) seeking expert advice from bioethicists and social scientists, (2) conducting qualitative and/or quantitative primary research, and (3) performing secondary research that includes published literature on social and ethical issues. At the HTA agency level, consultative mechanisms have also been recommended as a form of informing on research priorities and helping address the perceptions and expectations of lay members or representatives of stakeholder groups (e.g., patient associations).

In terms of the means for integrating ethical and social issues into HTA reports, bioethics expert advice may be sought and integrated into the HTA report, alternatively a specific group of experts may be mandated to produce a stand-alone report that complements the HTA report. In both these scenarios, ethics experts act as consultants. Some issues or principles can be generalized, however, country specificities are relevant, and analyses performed in one country or region may not be applicable in others. Thus, HTA agencies benefit from using local social scientists and ethicists who could become familiarized with the local HTA aims, methods, and constraints [14].

Legault and colleagues identified on HTA literature relating to the barriers to the incorporation of ethics in HTA nine ethical approaches: principlism, casuistry, coherence analysis, wide reflective equilibrium, axiology, the socratic approach, the triangular model, constructive technology assessment and social shaping of technology [10].

For the purpose of ethical evaluation based on the concept of a human person four principles have been identified: “a. the defense of human physical life; b. the contextual exercise of freedom and responsibility within the decision-making process; c. the safeguard of the therapeutic principle, according to which the human person has to be treated as a whole; d. the principles of sociality and subsidiarity, for which public and private authorities are called to help all persons in need” [10].

The EUnetHTA Core Model described the three steps of the Triangular model [27].

The first step of the Triangular Model is an in-depth study of all factual data concerning the technology, including its components, origin, purpose and consequences. The second step will be the ethical/anthropological analysis, in which the four evaluation principles mentioned above are applied to the case. Although not explicitly specified, it may be assumed that the analysis will comprise the evaluation of the consequences of the technology for the human being. The third step is the normative level and the ethical/anthropological analysis will lead the final ethical evaluation that normatively will determine a given real-world choice [10].

Globally, the differences between the different approaches identified are primarily related with their disciplinary foundation (rooted in philosophy, philosophy/theology, or sociology). Their complexity can be observed in the particular characteristics of the ethical assessment originating from their differing disciplinary foundation [10].

Assasi and colleagues also identified multiple guidance documents for the incorporation of ethical considerations in HTA, varying in their philosophical approach, structure and comprehensiveness.

This group noted ethical guidance documents have been designed for different purposes throughout the HTA process [25].

The ethical guidance documents identified frequently promoted the combination of normative reflection with descriptive approaches to the analysis of values and preferences of different stakeholders. However, the nature of the proposed procedural approaches varied considerably. They ranged from the approaches that advised on a general way of thinking about how to approach the assessment of ethical issues in HTA to those that provided analytical tools or case studies to aid users to understand a particular ethical analysis method [25].

The barriers to the implementation of ethical analysis in to HTA, originated from the very nature of ethical analysis itself are, as identified by Assasi and colleagues: the diversity in the types of ethical analysis proposed and their complexity and the difficulty of applying ethical analysis in such a way as to obtain concrete outcomes [10, 28].

Notwithstanding the advances achieved in this field, the majority of the HTA agencies apply ad hoc solutions in terms of the integration of ethics analysis in HTA and few systematically included ethicists in the procedure. Therefore, although the consideration of social and ethical implications of health technology is an unambiguous part of the HTA process, it is still not systematically performed, and the methods available for assessing ethical implications of health technology could be further developed [24].

In summary, to fully integrate ethics in HTA, several methods have been developed which may be useful for emphasizing ethical value judgments in the HTA process. At a higher level, the value base of a new technology can be considered as important as its evidence base and core values may be elaborated for HTA, namely universal access, freedom of choice, and quality care. Additionally, high-level reflections on the general values of health care (health, wellbeing, welfare) and its philosophical foundations, such as equity, equality, maximization of health or preferences or social values are required and should be more transparently used [24].

## Conclusions

Currently, several innovative gene therapies in development will address the underlying root cause of genetic diseases. This curative potential represents a paradigm shift in the approach of many life-threatening diseases.

In terms of the marketing authorization of ATMPs, a harmonized regulatory framework has been established in the EU. However, it will be important to promote consistent methods for conditional approval based upon the coverage with evidence or risk sharing. Subsequently, after the new treatment is made available for patients, it will be important to implement the appropriate arrangements for the collection and submission of supplementary real-world evidence and perform the corresponding review of the new evidence collected.

In terms of HTA the decisions remain at local level, which by itself is a source of inequality between EU citizens, namely in terms of access to the latest potentially lifesaving therapies, such as ATMPs.

ATMPs, may offer one-time, potentially curative treatments but, due to their characteristics, they will come to the market without solid evidence of their long-term effects and with a high price. These factors create important challenges for their reimbursement and adoption.

It seems clear that in an environment of limited healthcare budgets and increased scrutiny over the value preposition of any new product, it will be critical for developers to adopt an economic perspective early in the product development, from the pre-clinical stage, to ensure market access and long-term market viability.

It has been identified that one way to approach the specificities of ATMPs in terms of market access would imply broadening the definition of value to be able to systematically capture elements of value not captured in the QALY.

It seems critical to reach a consensus on how to appropriately reward value created by these new therapies that will allow to incentivize the appropriate risk taking and investments by their developers.

It appears has a logical solution that higher cost-effectiveness thresholds would support a higher value-based price and consequently a more appropriate reward for developers.

To achieve this purpose, it will be necessary to develop new methods that more truthfully capture the value of ATMPs that may cure and not just treat a particular disease.

Traditionally, HTA bodies have implemented higher cost-effectiveness thresholds for rare and life-threatening diseases. This is assumed to be in line with the wishes of the citizens of each country to agree to pay more for health gains in these particular situations. However, this willingness to pay will surely be further defied with the approval of more one-time gene therapies that are currently under development.

Several financing programs have been considered to address concerns about value and affordability, namely outcomes-based agreements, to reinsure payers regarding the effectiveness and long-term effects issues. However, even if the proposed costs for ATMPs are set inside the range of the cost-effectiveness thresholds defined, payers may not have the financial capability to support the access to these emerging groundbreaking products and assuring the affordability for these products since the early development is crucial.

An element that cannot be left aside from this discussion is the current growing role of patients in influencing the policy decisions taken and early patient engagement is key to attaining market access goals.

Globally, in the EU, in a setting where the equity between countries is promoted, access and equity in the field of ATMPs should be pursued. Policies should promote the equal access of EU citizens to the new innovative and potentially curative treatments. For this, the implementation of innovative approaches is required to assess the true value and fair price of ATMPs, assuring affordability and equity.

We are currently in a time of profound societal changes occurring in relation to healthcare technology development due to the emergency of groundbreaking therapies such as ATMPs. The HTA and market access decisions made in the scope of ATMPs raise important ethical questions and have important effects and consequences in society and therefore it is imperative that they are also assessed from an ethical perspective. In this context, HTA bodies have a greater responsibility to inform payers and the public on the reasons for the financing decisions made. For this, an effort should be made to make the ethical dimensions of HTA more transparent.

A complex aspect to face is the question of how to address value judgments in the scope of HTA. As it has been argued, value judgments are at the core of HTA, being an intrinsic part of the entire process.

It should be emphasized that to promote the ethical analysis in HTA, experts from different arenas such as HTA and bioethics, as well as health care professionals and patient representatives, should cooperate to further develop the methodology of reviews of normative ethical guidance to support evidence-based financing decisions. Particularly, for this integration to occur reliable methods should be used.

Ultimately, it should be accepted that even if it is not possible to agree on one procedure and approach in ethics applied to HTA, it is always possible to find a set of questions that are relevant in handling value judgment issues with respect to HTA.

The assurance that solid ethical standards are followed is intrinsic to science and research. On another perspective, science relies on social agreement and legal frames. Ideally, there should be no core conflict between ethical requirements and HTA in a scenario where the ultimate goal is to promote equity and access of patients to truly innovative therapies such as ATMPs, while assuring the sustainability of healthcare systems.
